# Isolation and characterization of reverse transcriptase fragments of LTR retrotransposons from the genome of *Chenopodium quinoa* (Amaranthaceae)

**DOI:** 10.1007/s00299-013-1468-4

**Published:** 2013-06-11

**Authors:** Bozena Kolano, Edyta Bednara, Hanna Weiss-Schneeweiss

**Affiliations:** 1 Department of Plant Anatomy and Cytology, University of Silesia, Jagiellonska 28, 40-032 Katowice, Poland; 2 Department of Systematic and Evolutionary Botany, University of Vienna, Rennweg 14, Vienna, Austria

**Keywords:** *Chenopodium quinoa*, In situ hybridization, Reverse transcriptase, Ty1-*copia* and Ty3-*gypsy* retrotransposons

## Abstract

****Key message**:**

**High heterogeneity was observed among conserved domains of reverse transcriptase (** ***rt*** **)** **isolated from quinoa. Only one Ty1-** ***copia rt*** **was highly amplified. Reverse transcriptase sequences were located predominantly in pericentromeric region of quinoa chromosomes.**

**Abstract:**

The heterogeneity, genomic abundance, and chromosomal distribution of reverse transcriptase (*rt*)-coding fragments of Ty1-*copia* and Ty3-*gypsy* long terminal repeat retrotransposons were analyzed in the *Chenopodium quinoa* genome. Conserved domains of the *rt* gene were amplified and characterized using degenerate oligonucleotide primer pairs. Sequence analyses indicated that half of Ty1-*copia rt* (51 %) and 39 % of Ty3-*gypsy rt* fragments contained intact reading frames. High heterogeneity among *rt* sequences was observed for both Ty1-*copia* and Ty3-*gypsy rt* amplicons, with Ty1-*copia* more heterogeneous than Ty3-*gypsy*. Most of the isolated *rt* fragments were present in quinoa genome in low copy numbers, with only one highly amplified Ty1-*copia rt* sequence family. The *gypsy*-like RNase H fragments co-amplified with Ty1-*copia*-degenerate primers were shown to be highly amplified in the quinoa genome indicating either higher abundance of some *gypsy* families of which *rt* domains could not be amplified, or independent evolution of this *gypsy*-region in quinoa. Both Ty1-*copia* and Ty3-*gypsy* retrotransposons were preferentially located in pericentromeric heterochromatin of quinoa chromosomes. Phylogenetic analyses of newly amplified *rt* fragments together with well-characterized retrotransposon families from other organisms allowed identification of major lineages of retroelements in the genome of quinoa and provided preliminary insight into their evolutionary dynamics.

## Introduction

Mobile genetic elements are ubiquitous and abundant components of nearly all eukaryotic genomes. They are divided into two main groups: DNA transposons (class I) and retroelements (class II; Flavell et al. 1992; Kumar and Bennetzen 1999; Friesen et al. 2001; Du et al. 2010; Tenaillon et al. 2010). Class II retroelements are particularly abundant in plant genomes. They transpose replicatively to new genome locations via RNA intermediates reversely transcribed into DNA prior to their integration into the host genome (Wicker et al. 2007). Many plant genomes accumulate large amounts of mobile genetic elements mainly in the dispersed repetitive DNA fraction (Hawkins et al. 2006). The copy number of retrotransposons may vary even among closely related plant taxa, e.g., transposable element content in *Zea luxurians* is 1.35-fold greater than in *Zea mays* (Tenaillon et al. 2011). Retrotransposons can further be divided into two groups depending on the presence/absence of long terminal repeats (LTRs). Within the LTR retrotransposons, two subclasses, Ty1-*copia* and the Ty3-*gypsy*, are particularly abundant and well analyzed in plants. These two types differ in gene order within the *pol* domain that encodes protease, RNase H, reverse transcriptase (*rt*), and integrase. The organization of the coding domains in the Ty3-*gypsy* elements is similar to that of retroviruses (although they often lack putative envelope coding genes), while in the Ty1-*copia* elements the integrase domain is found upstream of the *rt* domain (Kumar and Bennetzen 1999; Bennetzen 2000). It is generally accepted that retrotransposons affect genome size, organization, and function (Parisod et al. 2009). Not only can they substantially change genome size because of their ongoing replicative mode of transposition (and usually slower rate of removal), but they can also generate mutations by inserting new copies within or near gene(s), or cause chromosomal rearrangements via illegitimate or unequal recombination (Bennetzen 2000; Hawkins et al. 2006; Parisod et al. 2009).

The *rt* gene of the retroelements has several conserved domains characteristic for individual retroelement families (Xiong and Eickbush 1990; Peterson-Burch and Voytas 2002). Availability of degenerate oligonucleotide primers complementary to the conserved regions of the *rt* has allowed amplification of *rt* fragments and sampling retrotransposon diversity in various plant genomes (Brandes et al. 1997; Friesen et al. 2001). Many phylogenetic comparisons of populations of retrotransposons, particularly Ty1-*copia*-like elements, have been performed, both within and among related groups of taxa (Brandes et al. 1997; Heslop-Harrison et al. 1997; Pearce et al. 2000; Sant et al. 2000; Friesen et al. 2001; Navarro-Quezada and Schoen 2002; Alix et al. 2005; Park et al. 2007; Ma et al. 2008; Parisod et al. 2012). Ty3-*gypsy*-like elements have also been analyzed in several plant groups although less extensively than Ty1-*copia* (Suoniemi et al. 1998; Friesen et al. 2001; Alix et al. 2005; Hill et al. 2005; Park et al. 2007; Ma et al. 2008). Varying patterns of chromosomal distribution of individual retroelement families, ranging from dispersed to localized near-centromeric, were documented in plants using fluorescent in situ hybridization (FISH) (Brandes et al. 1997; Belyayev et al. 2001; Friesen et al. 2001).

Quinoa (*Chenopodium quinoa* Willd) is one of the most important food crops in the Andean region of South America. Its grain has an excellent balance of carbohydrates, lipids, and proteins with essential amino acid compositions optimal for human nutrition (Popenoe et al. 1989). *Chenopodium quinoa* is an allotetraploid species with 2*n* = 4*x* = 36 chromosomes and relatively small genome (1.487 pg/1C or 1,453.8 Mb; Kolano et al. 2012), which shows disomic inheritance for most qualitative traits (Ward 2000). Although recent studies of the quinoa genome have provided some insight into its origin and composition of its repetitive genome fraction (Mason et al. 2005; Maughan et al. 2006; Kolano et al. 2008, 2011), retrotransposable elements were not analyzed so far.

The present work aims to characterize the diversity of *rt* fragments of Ty1-*copia* and Ty3-*gypsy* retrotransposons from the *C. quinoa* genome amplified and cloned using degenerate primers. It also attempts to examine their heterogeneity, phylogenetic relationships, abundance in the genome, and chromosomal organization.

## Materials and methods

### Plant material and isolation of DNA

Seeds of *C. quinoa* cv. Tango were obtained from Dr. Susanne Dobler (Albert-Ludwigs University, Freiburg, Germany). Plants were grown in pots in a greenhouse under a 16 h photoperiod at 19 ± 2 °C. Total genomic DNA was extracted from young leaves using the standard CTAB method (Doyle and Doyle 1987).

### Polymerase chain reaction (PCR) and cloning of PCR products

The *rt* domains of *copia* and *gypsy* retrotransposons were amplified from the genomic DNA of quinoa using PCR with degenerate primers. The degenerate primer pairs were used to amplify *rt* domains of Ty1-*copia* (Flavell et al. 1992) and Ty3-*gypsy* elements (Friesen et al. 2001). PCR amplification was carried out in GeneAmpPCR System 9700 (Applied Biosystems, USA). The reaction mixture contained 60 ng DNA, 25 pmol of each primer (Genomed, Warsaw, Poland), 0.2 mM of each dNTPs (Genomed), 1× buffer (including 3.5 mM MgCl_2_) and 1 U of GoTag polymerase (Promega, Madison, USA). The PCR program consisted of initial denaturation at 94 °C for 3 min, followed by 30 cycles at 94 °C 1 min, 1 min at 45 °C, and 1 min at 72 °C, with a final elongation step of 5 min at 72 °C. PCR products were purified from the gel using the Qiaquick Gel Extraction Kit (Qiagen, Germany) and cloned into pGEM-T Easy vector (Promega) following the manufacturer’s instructions. Randomly chosen recombinant colonies were selected for plasmid DNA isolation using a standard mini-prep method (Sambrook et al. 1987). Two independent rounds of PCR amplification and cloning were carried out for both *copia* and *gypsy* elements. The presence of inserts of desired length was verified by PCR with M13 primers (Park et al. 2007). The cloned fragments were sequenced in both directions using M13 universal primers and BigDye terminator v3.1 Cycle Sequencing Kit technology (Applied Biosystems) in 3730xl DNA Analyzer (Applied Biosystems). Clone names are composed of an abbreviated taxon name, an abbreviation for the type of element (ty for *copia* and gy for *gypsy*) and the clone serial number. For example, pCquty1 stands for the Ty1-*copia*-type clone number 1 from *C. quinoa*.

### Dot blot and hybridization

The genomic abundance of selected *rt* fragments of Ty1-*copia* and Ty3-*gypsy* retrotransposons was determined using dot blot hybridization. Plasmids containing clones of *rt* were purified with the QIAprepMiniprep Kit (Qiagen), quantified (NanoDrop, TermoScientific, USA) and adjusted to a final concentration of 20 ng/μl. Thirty microlitres of each cloned sequence was denatured at 96 °C for 10 min, and transferred onto a positively charged nylon membrane (Roche, Switzerland) using the Dot Blot 96 System (Biometra, Germany). DNA was fixed to the membrane by UV treatment, washed with sterile water, and air-dried. Genomic DNA of *C. quinoa* cv Tango labeled with digoxigenin (DIG Nick Translation Kit, Roche) was used as the DNA probe. Hybridization was performed at 40 °C according to the manufacturer’s instructions using the DIG High Prime DNA Labelling and Detection Starter Kit II (Roche). High stringency washes were performed to retain only hybridization signals at the level of high sequence similarity (90 %: 0.1× SSC and 0.1 % SDS at 68 °C). Hybridization signals were documented and quantified using ChemiDocXRS (BioRad, USA).

Dot blot hybridization was also used for the estimation of copy number of the abundant pCquty119 clone. The genomic DNA samples and clone pCquty119 were denatured in 0.4 M NaOH/0.01 M EDTA for 10 min at 98 °C and transferred to a positively charged nylon membrane (Roche) using a vacuum blotter. Clone pCquty119 labeled with digoxigenin was used as the DNA probe. Hybridization was performed using the DIG High Prime DNA Labelling and Detection Starter Kit II (Roche) as described above. Integrated densities used for the calculation of copy number were obtained using ImageJ software (Schneider et al. 2012).

### Sequence analysis

Cloned sequences were checked against GenBank database for their homology to previously characterized plant retroelement lineages. DNA sequences were manually aligned in BioEdit version 7.1.3.0 (Hall 1999) and the alignment was guided by conserved amino acid domains of Ty1-*copia* and Ty3-*gypsy* plant retrotransposons (Xiong and Eickbush 1990). Gaps were introduced to retain open reading frames (ORFs), and primer-binding regions were excluded prior to the analyses. Genetic similarities were calculated on both nucleotide and amino acid levels using p-distances in MEGA4 (Tamura et al. 2007). The translated amino acid sequences of quinoa *rt* sequences were compared with elements isolated from related genera (*Amaranthus*, *Beta*, and *Spinacia* species) as well as with previously characterized elements representing main evolutionary lineages of plant Ty1-*copia* retrotransposons (available in GenBank and GrainGenes 2 database; Wicker and Keller 2007; Llorens et al. 2009). Neighbor-joining (NJ) analyses of the nucleotide data sets were conducted using p-distance in MEGA 4. Pairwise deletion of missing data (gaps) was used to compute the distance matrices. Nodal support was accessed via bootstrapping using 1,000 bootstrap (BS) replicates.

### Chromosome preparation and fluorescence in situ hybridization

Young leaves of quinoa were pretreated with 2 mM 8-hydroxyquinoline for 4 h at room temperature, fixed in methanol:glacial acetic acid (3:1) and stored at −20 °C until use. Metaphase chromosome spreads were prepared as described earlier (Kolano et al. 2011). The clones containing *rt* inserts were labeled with digoxigenin-11-dUTP using PCR with universal M13 primers (Hajdera et al. 2003). Fluorescence in situ hybridization was preformed according to the protocols described by Schwarzacher and Heslop-Harrison (2000) and Kolano et al. (2011). Briefly, the hybridization mixture consisting of 100 ng of labeled DNA probe, 50 % formamide, 2× SSC, 10 % dextran sulfate, 0.1 % SDS, and 0.3 μg/μl of blocking DNA was denatured for 10 min at 85 °C, and applied to chromosome preparations. The slides and hybridization mixture were denatured together at 72 °C for 5 min in an in situ Thermal Cycler (ThermoHybaid, Franklin, USA) and allowed to hybridize in a humid chamber at 37 °C for 72 h. Stringent washes (two times in 0.1× SSC at 42 °C) were followed by detection of digoxigenin using FITC-conjugated primary anti-digoxigenin antibody (Roche). Signal was amplified with FITC-conjugated anti-sheep secondary antibody (Jackson ImmunoResearch, Suffolk, UK). Preparations were mounted in antifade solution Vectashield (Vector Laboratories, Peterborough, UK) containing 2 μg/ml of DAPI (4′,6-diamidino-2-phenylindole).

## Results

### Isolation and sequence characterization of *rt* fragments

PCR with degenerate primers designed to amplify conserved domains of the *rt* gene of Ty1-*copia* (Flavell et al. 1992) and Ty3-*gypsy* retrotransposons (Friesen et al. 2001) resulted in fragments of expected length (c. 260 and 420 bp, respectively). The first round of PCR and cloning yielded 39 sequences of Ty1-*copia rt* and 43 sequences of Ty3-*gypsy rt* and the second independent round of PCR and cloning yielded 33 Ty1-*copia rt* clones and 32 Ty3-*gypsy rt* clones. In total, 72 clones of Ty1-*copia rt* and 75 clones of Ty3-*gypsy rt* with homology to known retroelements (GenBank) were selected for further analysis. The sequences are deposited in GenBank under accession numbers: JN575483–JN575554 (Ty1-*copia*) and JN594743–JN594817 (Ty3-*gypsy*).

Isolated Ty1-*copia rt* fragments ranged from 252 (pCquty86, pCquty46, pCquty37) to 305 bp (pCquty122) in length, most fragments being 267 bp (26 %) long (Table 1). The Ty3-*gypsy rt* sequenced ranged from 393 bp (pCqugy71) to 421 bp (pCqugy51) in length, with most abundant variants of 416–417 bp (70 %; Table 1). The two shortest clones, pCqugy71 (393 bp) and pCqugy126 (396 bp), lacked primer-binding sites encoding peptide YAKLSKC. Both Ty1-*copia* and Ty3-*gypsy rt* sequences were AT-rich with average of 60 % AT content. The putative *rt* sequences were translated into amino acids and alignment corrected for frame shifts were necessary to maintain an ORF. Thirty-five out of 72 Ty1-*copia rt* sequences (49 %) contained premature stop codons and/or indels disrupting the reading frame, while the remaining 37 sequence fragments (51 %) were potentially functional. Among the Ty3-*gypsy rt* sequences, 29 clones (39 %) possessed intact reading frames while 46 clones (61 %) had disrupted reading frames. Alignment of putative amino acid sequences of the clones revealed some variation in their translated primer sequences, reflecting the heterogeneous nature of these sequences in the quinoa genome, which may impact the pool of fragments amenable for amplification with degenerate primers.

**Table 1:** Analyzed *rt* sequence length in bp (range/variants more common than 25 %, including primer regions), number of clones with intact open reading frames (ORFs) and sequence similarity [given as (minimum) average (maximum)] of *rt* fragments of Ty1-*copia* and Ty3-*gypsy* isolated from the *Chenopodium quinoa* genome

	Number	Clones with intact ORF	Length	Similarity
Ty1-*copia*	72	37	252–305/267 (26 %)	(36) 58 (100)
*Tork/TAR*	30	18	252–270/267 (60 %)	(54) 77 (100)
Subclade A-1	20	17	252–267/267 (75 %)	(89) 96 (100)
Subclade A-2	4	0	265–267/266 (50 %)	(85) 90 (97)
*Tork/Angela*	2	2	264	100
*Oryco/Ivana*	1	1	261	–
*Retrofit/Ale*	39	15	254–306/261&264 (51 %)	(50) 57 (100)
Subclade B-1	4	4	264	(91) 94 (98)
Ty3-*gypsy*	75	29	393–421/417 (50 %)	(58) 70 (100)
*Del/Tekay*	64	28	400–421/417 (55 %)	(60) 74 (100)
Subclade A-1	10	5	415–417/417 (50 %)	(89) 93 (98)
Subclade A-2	4	2	416–417/417 (50 %)	(88) 91 (94)
Subclade A-3	6	2	416–421/417 (67 %)	(82) 92 (95)
Subclade A-4	9	6	415–417/417 (89 %)	(89) 93 (100)
Subclade A-5	8	4	406–417/417 (62 %)	(86) 92 (98)
Subclade A-6	6	3	414–417/417 (67 %)	(97) 87 (84)
Subclade A-7	5	1	413–417/417 (40 %)	(80) 86 (92)
Subclade A-8	13	5	401–419/417 (38 %)	(78) 83 (96)
*Reina*	11	1	393–413/416 (27 %)	(60) 68 (100)
Subclade B-1	7	1	416–417/416 (43 %)	(69) 77 (100)

High nucleotide heterogeneity was observed among isolated *rt* fragments. The average sequence diversity among Ty1-*copia rt* elements reached 42 %, with the maximum diversity of 64 % (Table 1). Only four pairs of identical sequences (pCquty46 and pCquty37; pCquty85 and pCquty82; pCquty104 and pCquty105; pCquty119 and pCquty118) as well as a group of three identical sequences (pCquty102, pCquty108, and pCquty125) were found. The Ty3-*gypsy rt* fragments revealed lower sequence heterogeneity compared to Ty1-*copia*, as evidenced by both significantly lower maximum (42 %) and average nucleotide diversity (30 %; Table 1). Only two pairs of identical *gypsy* *rt* sequences were found (pCqugy46 and pCqugy49; pCqugy84 and pCqugy120). Analyses of the amino acid heterogeneity of the amplified *rt* fragments also revealed higher diversity among Ty1-*copia rt* fragments than among *gypsy* (Ty1-*copia*: max 63 %, average 45 %; Ty3-*gypsy*: max 47 %, average 26 %).

### Phylogenetic analysis of quinoa Ty1-*copiart* clones

**Fig. 1:**
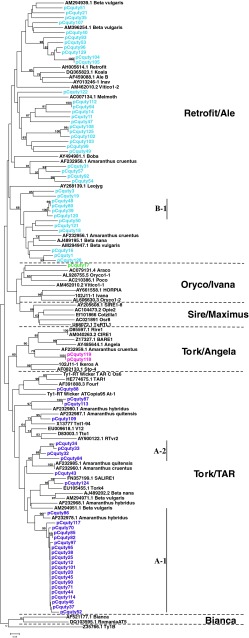
Phylogenetic relationships of Ty1-*copia rt* fragments of *C. quinoa* and other plants’ genomes based on translated nucleotide sequence analyses. *Numbers* *above* *branches* indicate bootstrap support (BS) above 60 %. *Scale bar* indicates genetic distance. The quinoa *rt* sequences from *Retrofit/Ale* lineage are labeled with *blue*, the quinoa *rt* fragment from *Oryco/Ivana* lineage with *green*, elements from *Tork/Angela* lineage with *pink* and quinoa sequences from *Tork/TAR* with *violet*. *A-1*, *A-2*, and *B-1* represent of subclades of very similar quinoa *rt* sequences

**Fig. 2:**
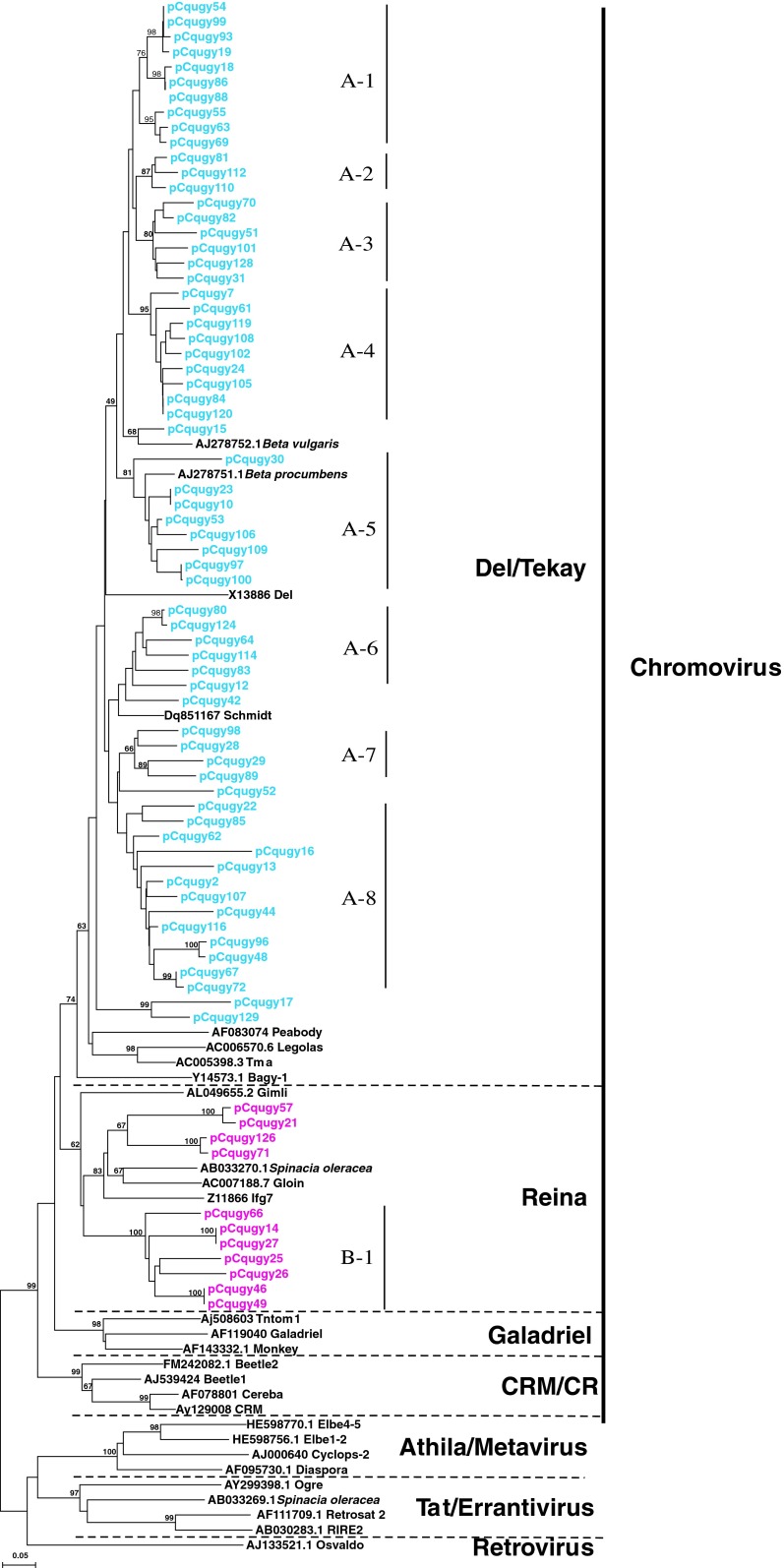
Phylogenetic relationships of Ty3-*gypsy rt* fragments amplified from of *C. quinoa* and other plants’ genomes based on translated nucleotide sequence analyses. *Numbers above branches* indicate bootstrap support above 60 %. *Scale bar* indicates genetic distance. The quinoa *rt* sequences from *Del/Tekay* lineage are labeled with *blue* and quinoa elements from *Reina* lineage with *pink*, *A-1* to *A-8* and *B-1* indicate subclades of very similar quinoa *rt* sequences

Newly amplified sequences of quinoa *copia*-type *rt* clones were aligned with *rt* fragments reported from related genera (*Amaranthu*s and *Beta*; GenBank; Fig. 2) as well as with previously characterized elements representing main recognized evolutionary lineages of plant Ty1-*copia* retrotransposons (available in GenBank and GrainGenes 2 database; Wicker and Keller 2007; Llorens et al. 2009). The results of phylogenetic analyses allowed identification of major lineages of Ty1-*copia* amplified from quinoa genome. Comparison of the amino acid sequence of *rt* of quinoa elements with other Ty1-*copia* retrotransposons revealed that genome of quinoa harbors at least four major *copia* lineages described by Wicker and Keller (2007; Fig. 1). The most numerous group (39 elements) of quinoa *rt* fragments represents a *Retrofit/Ale* lineage. Quinoa *rt* fragments from this lineage showed average sequence similarity of 57 % (Table 1). In *Retrofit/Ale* lineage, only one small subclade (B-1), containing four quinoa sequences with relatively high sequence similarity (91–98 %) (Table 1; Fig. 1), was recovered, while the remaining sequences were more heterogeneous. Lineage *Tork/TAR* comprised 30 sequences with average sequence similarity of 77 % (Table 1; Fig. 1). Within this lineage, 20 quinoa sequences (subclade A-1) shared similarity of 89–100 % and most of these (17 clones) possessed intact reading frames. The second subclade (A-2) consisted of four quinoa elements (sequence similarity of 85–97 %) all with disrupted reading frames (Table 1). Two quinoa *rt* fragments belonged to the *Tork/Angela* lineage and only one quinoa clone (pCquty17) exhibited similarity to *Oryco/Ivana* retrotransposons (Table 1; Fig. 1).

The major lineages of *copia* elements previously amplified from *Beta* and/or *Amaranthus* species (Fig. 1) were also found in quinoa genome. Sequences from subclade A-1 share 83–86 % similarity to *Amaranthus* sequence AF232978.1. Other sequences from the A-2 clade showed 77–85 % similarity to two *Amaranthus* elements (AF232960.1; AF232985.1). Clone pCquty124 exhibited 84 % nucleotide sequence similarity to a corresponding region of SALIRE1 retrotransposon from *Beta vulgaris*.

### Phylogenetic analysis of quinoa Ty3-*gypsyrt* clones

Newly amplified sequences of quinoa Ty3-*gypsy* *rt* fragments were aligned to *rt* fragments reported from related genera (S*pinacia* and *Beta* species; Kumekawa et al. 1999; Gindullis et al. 2001), as well as elements representing main evolutionary lineages of plant Ty3-*gypsy* retrotransposons (Llorens et al. 2009). The dendrogram based on amino acid sequence alignment allowed assignment of quinoa *rt* fragments to *Del/Tekay* and *Reina* of the *Chromovirus* lineage (Fig. 2). Most of the isolated quinoa elements represented the *Del/Tekay* lineage. This evolutionary lineage contained 64 quinoa *rt* fragments (85 %) with average sequence similarity of 74 % (range 60–100 %; Table 1). *Reina* was represented by 11 quinoa elements with average sequence similarity of 68 % (range 60–100 %; Table 1). The phylogenetic tree of Ty3-*gypsy* *rt* sequences was more structured than the one based on Ty1-*copia rt* fragments. In *Del/Tekay* lineage eight subclades (A-1 to A-8), containing quinoa sequences with relatively high average sequence similarity (83–93 %) (Table 1; Fig. 2) were recovered. In *Reina* lineage only one subclade (B-1), containing seven quinoa sequences with average sequence similarity 77 % (Table 1; Fig. 2) was recovered. Nearly all Ty3-*gypsy rt* fragments with intact reading frames belonged to *Del/Tekay* lineage. All quinoa fragments from *Reina* lineage, with the exception of clone pCqugy66, represented potential pseudogenes (possessed stop codons or frameshifts).

Some of the previously reported Ty3-*gypsy rt* clones from *Beta* showed high similarity to quinoa elements (Fig. 2), including sequences AJ278752.1 (*B. vulgaris)* and AJ278751.1 (*Beta procumbens*) with 72–80 % similarity to the quinoa elements from *Del/Tekay* lineage. Schmidt retrotransposon (*B. vulgaris*) showed 70–74 % similarity to quinoa elements from part of *Del/Tekay* lineage.

### Relative abundance in the genome and chromosomal localization of *copia* and *gypsy rt* fragments

**Fig. 3:**
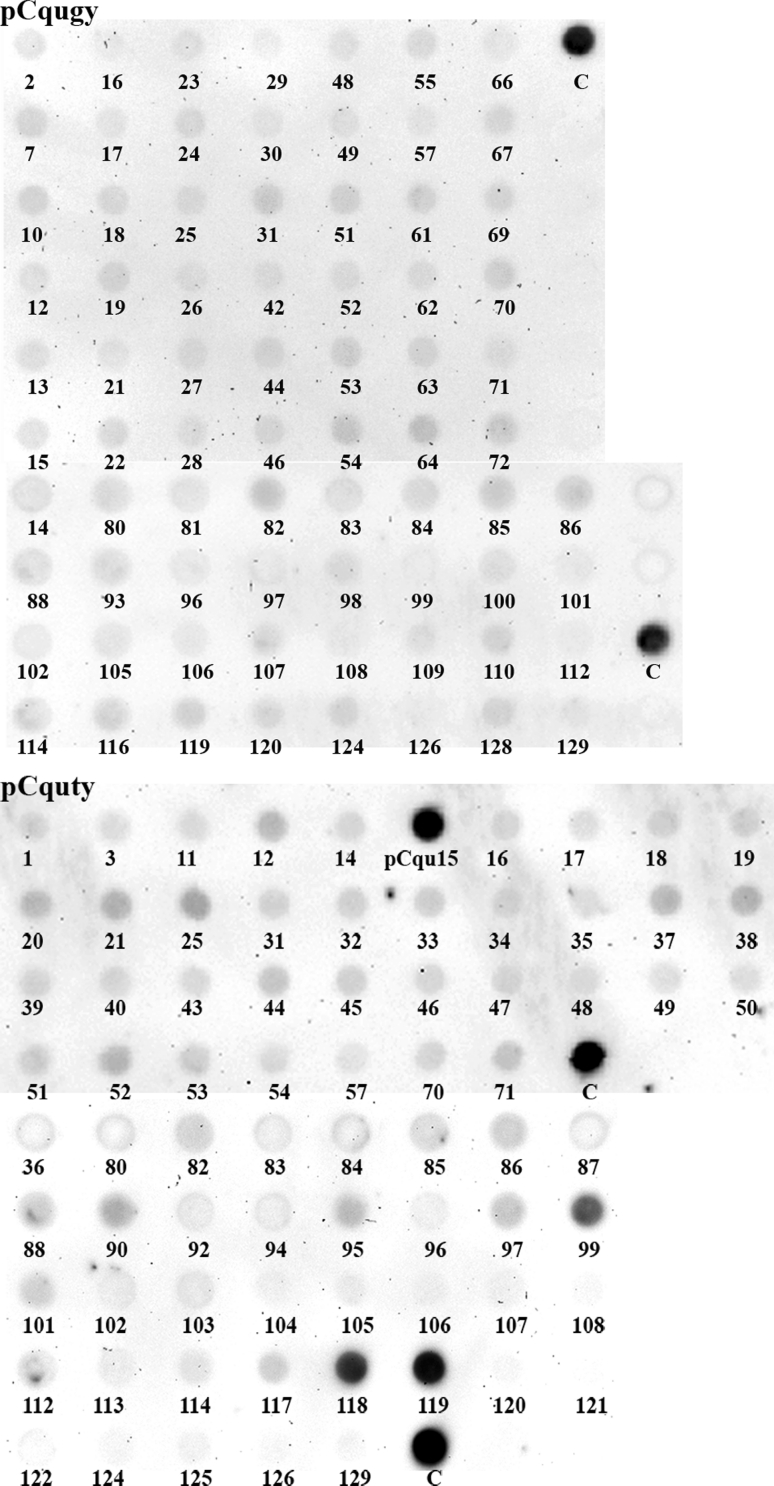
Relative abundance of Ty1-*copia* and Ty3-*gypsy* elements in the *C. quinoa* genome. Dot blot hybridization to Ty1-*copia rt* and Ty3-*gypsy rt* clones isolated from *C. quinoa* using genomic DNA of *C. quinoa* as a probe. *Numbers* indicate the clone numbers. *C* positive control (genomic DNA of *C. quinoa*)

**Fig. 4:**
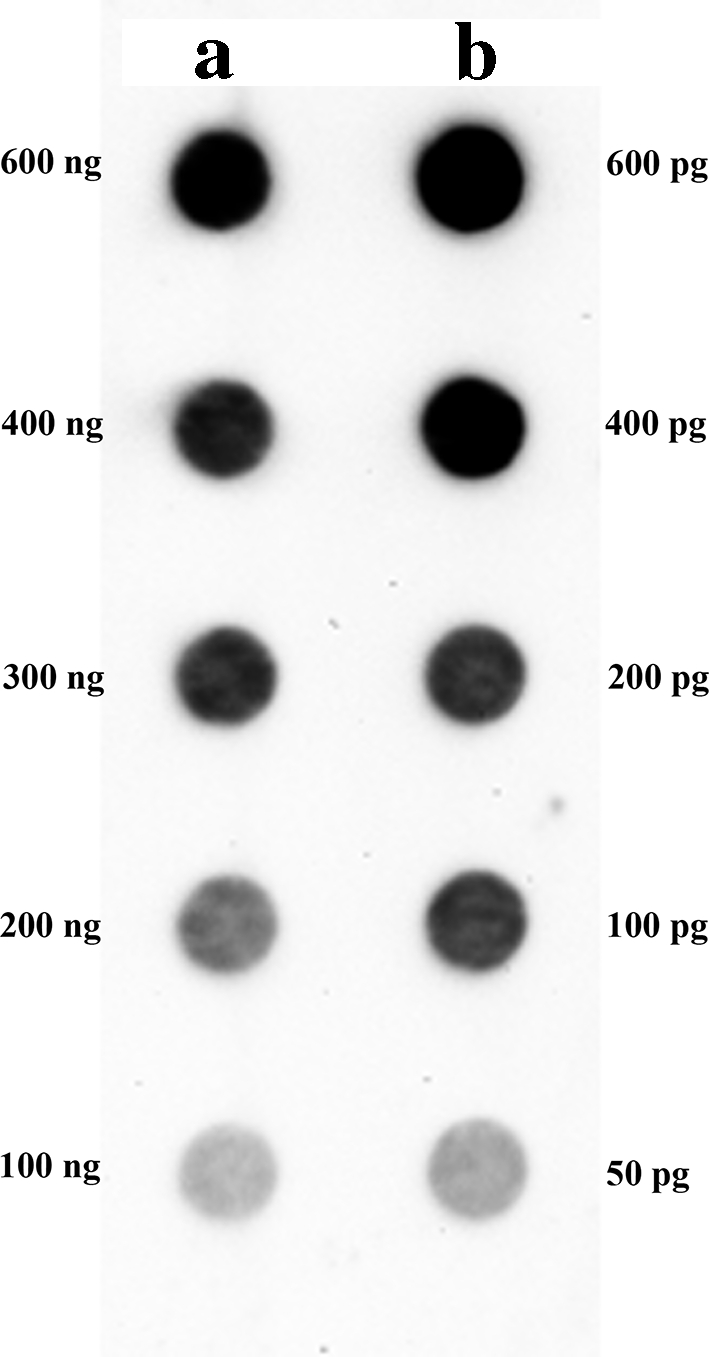
Dot blot used for the estimation of the copy number of clone pCquty119 in the genome of *C. quinoa.* Different amounts of genomic DNA of *C. quinoa* (*row a*) and serial dilutions of pCquty119 clone (*row b*) were dot blotted on a membrane. Labeled clone pCquty119 was used as a DNA probe

The relative abundance of isolated *rt* clones in the *C. quinoa* genome was estimated using dot blot hybridization. The dilutions of all isolated clones were used to compare their abundance (signal intensity) in the genomic DNA of *C. quinoa.* Very strong hybridization signals were observed for only two *copia rt* clones: pCquty118 and pCquty119 (clones with identical nucleotide sequences; Fig. 3), indicating their high abundance in the quinoa genome. The copy number of these clones in *C. quinoa* genome has been estimated as 2,600–3,000 molecules per haploid genome (1C DNA) (Fig. 4). A less intense hybridization signal was observed for the clone pCquty99. The remaining analyzed Ty1-*copia rt* clones showed only weak or very weak hybridization signals suggesting their lower copy numbers in the quinoa genome.

**Fig. 5:**
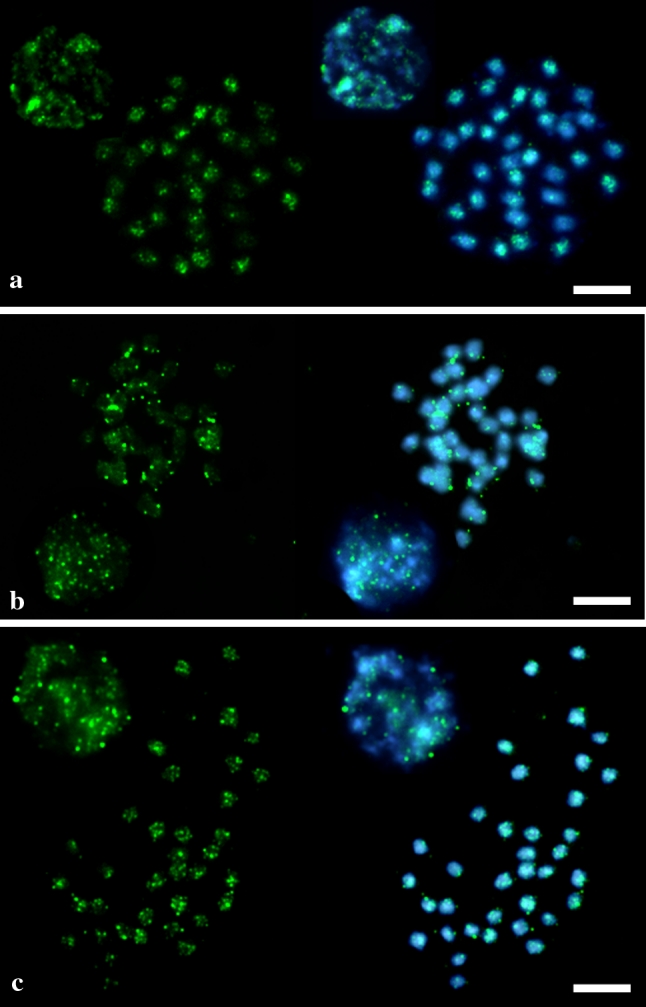
Fluorescence in situ hybridization of reverse transcriptase fragments (*green fluorescence*) to DAPI-counterstained metaphase chromosomes and interphase nuclei of *C. quinoa*: **a** clone pCquty119; **b** heterogeneous probe containing all Ty1-*copia rt* clones except for pCquty118 and pCquty119; **c** heterogeneous probe representing all Ty3-*gypsy rt* clones. *Scale bar* 5 μm

Dot blot hybridization of Ty3-*gypsy rt* clones did not reveal the presence of any strongly amplified clones, with all clones hybridizing only weakly to genomic DNA (Fig. 3). Physical distribution of Ty1-*copia* retrotransposons in the quinoa genome was analyzed in metaphase chromosomes and interphase nuclei. Clones which showed stronger hybridization signals in dot blot tests were used as probes for FISH [pCquty119 (identical with pCquty118) and pCquty99]. Hybridization with pCquty119 probe reveled clear signals in the chromosomes (Fig. 5a). Both the chromosomal distribution and intensity of the hybridization signals of pCquty119 differed among chromosomes. Approximately 16 (out of 36) chromosomes exhibited relatively strong hybridization signals, whereas the rest of the signals were very weak or signals were absent. pCquty119 was predominantly located in pericentromeric regions of the chromosomes and usually absent/undetectable from distal chromosome parts. Scattered hybridization signals were also observed in interphase nuclei, predominantly coinciding with heterochromatin (bright DAPI-stained regions). Hybridization with pCquty99 did not yield any detectable signals, indicating that the copy number of this sequence is below the detection limit of FISH.

Chromosomal localization of the Ty1-*copia* elements that appeared to be only weakly amplified in the quinoa genome (dot blot) was tested using the heterogeneous probe cocktail containing all isolated Ty1-*copia* clones except for the clones pCquty118 and pCquty119. FISH with such a cocktail revealed dispersed hybridization signals in pericentromeric and/or subterminal positions of all chromosomes (Fig. 5b). Hybridization signals observed in interphase nuclei predominantly coincided with heterochromatic regions, but also with some euchromatic regions.

Localization of Ty3-g*ypsy* retrotransposons in interphase nuclei and metaphase chromosomes of *C. quinoa* has been analyzed using the heterogeneous cocktail containing all isolated Ty3-*gypsy* clones as a probe. All of these clones were only weakly amplified in the quinoa genome (dot blot). FISH signals were observed in most metaphase chromosomes and showed a dispersed pattern with weak clustering, mostly in pericentromeric regions (Fig. 5c). Hybridization signals in the interphase nuclei predominantly co-localized with heterochromatic regions.

### Clones pCqu15 and pCqu22: sequence characterization and chromosomal organization

PCR amplification of Ty1-*copia rt* fragments using standard degenerate primers encoding for TAFLHG and YVDDML resulted in co-amplification of 13 clones with no homology to known *rt*. Among these, two clones (pCqu22 and pCqu15) were shown to be highly amplified in the *C. quinoa* genome (names of these clones have been simplified indicating the species name *Cqu* and serial number only). These two sequences were highly similar (92 %). pCqu22 was 279 bp long, whereas pCqu15 was 2 bp shorter. The alignment of putative amino acid sequences of these clones revealed presence of a complete upstream primer encoding the TAFLHG domain, whereas deletion of the first three nucleotides has occurred within the downstream primer region (YVDDML). Both sequences showed similarity to a fragment of RNase H of a Ty3-*gypsy* retrotransposon (blastx against GenBank database; BAE96746.1, AAK52571.1). The sequences are deposited in GenBank under accession numbers: KC869994 and KC869995.

**Fig. 6:**
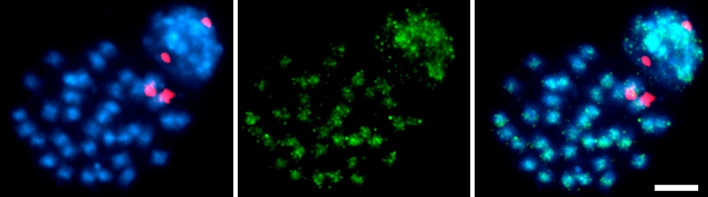
Fluorescence in situ hybridization of pCqu22 clone (*green fluorescence*) and 35S rDNA (NOR) (*red fluorescence*) to DAPI-counterstained metaphase chromosomes and interphase nuclei of *C. quinoa*. *Scale bar* 5 μm

Chromosomal organization of these two sequences was analyzed using FISH with the clone pCqu22. FISH signals were located mainly in the pericentromeric and centromeric regions of all chromosomes, but they varied in intensity. Some chromosomes possessed also faint signals of pCqu22 in more distal regions. This sequence type was absent from nucleolus organizing regions (NOR; Fig. 6). Hybridization signals in interphase nuclei mostly co-localized with DAPI-positive heterochromatic regions. The euchromatic regions of interphase nuclei showed fewer and fainter hybridization signals.

## Discussion

*Chenopodium quinoa* (Amaranthaceae) is a very important seed crop in South America. Although it is becoming an increasingly popular alternative plant crop also in the USA and Europe, its tetraploid genome origin, structure, and evolution are very poorly understood. Retrotransposons are a source of genetic diversity potentially causing changes in genome structure and gene expression, and are considered to be an important factor in genome plasticity and evolution (Kidwell and Lisch 1997; Ma et al. 2005; Hawkins et al. 2006; Zedek et al. 2010; Lisch 2013). To our knowledge, this study is the first to survey the diversity of LTR retrotransposons in the *C. quinoa* genome. Reverse transcriptase domains of two superfamilies Ty1-*copia* and Ty3-*gypsy* were amplified from the *C. quinoa* genome using degenerate primers. PCR amplification of heterogeneous templates typically suffers from bias toward some sequence types and underrepresentation of others. Such bias is further enhanced by the choice of degenerate primers. In an attempt to at least partly alleviate such methodological effects, two independent rounds of amplification and cloning were conducted and more than 70 clones of each retrotransposon superfamilies (*copia* and *gypsy*) were analyzed. Such an approach should have increased chances of obtaining more representative genomic sampling of analyzed retroelements and allow for broader sampling of their diversity in the *C. quinoa* genome. The second PCR round yielded only two new Ty1-*copia* element types, which did not belong to any strongly supported clades and no new Ty3-*gypsy* *rt* types. The results suggest that the obtained *rt* clones reasonably well represent the retroelement diversity in the quinoa genome (Park et al. 2007). One should, however, bear in mind that the degenerate primers were designed based on limited numbers of retrotransposon types and some lineages of retrotransposons might have been underrepresented in the amplification using those primers (Park et al. 2007).

Previous survey of LTR retrotransposons in land plants defined six major common evolutionary Ty1-*copia* lineages: *Tork/TAR, Tork/Angela*, *Sire/Maximus*, *Oryco/Ivana* and *Retrofit/Ale*, and *Bianca* (Wicker and Keller 2007; Llorens et al. 2009). *Sire/Maximus* and *Bianca* were the only Ty1-*copia* lineages not found among newly amplified quinoa *rt* fragments. *Oryco/Ivana*, the second lineage of the *Sireviruses* was represented in our dataset only by one clone. Among Ty3-*gypsy* elements, typically three major evolutionary lineages are distinguished in land plants (Llorens et al. 2009; Du et al. 2010): non-chromodomain LTR retrotransposons *Athila* and *Tat* and *Chromovirus* lineage. Within the latter several sublineages are commonly described: *Del/Tekay, Reina, Galadriel, CRM* (*Chromoviruses*). The majority of isolated quinoa Ty3-*gypsy rt* clones represented *Del/Tekay* lineage. No elements from the *Chromovirus* sublineages *Galadriel* and *CRM* and no clones similar to *Athila* or *Tat* lineages were found. Newly amplified quinoa *rt* fragments clearly were dominated by one Ty3-*gypsy* and two Ty1-*copia* evolutionary lineages. *Sireviruses* have been shown to proliferate quite extensively in many analyzed plant genomes often representing the majority of Ty1-*copia* elements of the genome [e.g., ~90 % of Ty1-*copia* complement, c. 20 % of the genome in maize (Bousios et al. 2012)]. Some retrotransposon lineages [Beetle1 (*CRM/CR*), Weber and Schmidt 2009; Cotzilla1 (*Sirevirus*), Weber et al. 2010; *Athila*, Wollrab et al. 2012], reported as highly amplified in the genome of *B. vulgaris*, a taxon closely related to *Chenopodium*, were not represented among amplicons from the *C. quinoa* genome. This might reflect real differences of the presence and abundance of different lineages (Du et al. 2010) in the quinoa genome, caused by their different evolutionary dynamics. Alternatively, it can indicate amplification bias caused by failure to amplify *rt* fragments that distinctly differ in primer-binding regions from the degenerate primers used. More meaningful comparisons would only be possible when the same methods were used to estimate the abundance of retroelement lineages in the above-mentioned genera.

Ty1-*copia rt* fragments amplified from the quinoa genome exhibited high levels of heterogeneity (average sequence heterogeneity of 42 %) in agreement with the data reported for various other angiosperm species: e.g., *Phelipanche* (Orobanchaceae; Park et al. 2007), or *Olea europaea* ssp. *sativa* (Oleaceae; Stergiou et al. 2002). Many of the elements (49 %) were not functional and possessed stop codons and/or frameshifts, and the accumulation of mutations indicated that they might be remnants of more ancient amplifications. Elements resulting from older activity cycles often experience deletions or fragmentation by illegitimate or unequal homologous recombination and are rich in mutations of methylated cytosine to thymine in the *rt* region (Vitte and Bennetzen 2006). On the other hand, evolutionarily younger transposition events often result in relatively homogeneous groups of retroelements, as most likely evidenced by members of subclade A-1 (Hill et al. 2005). This clade consists of 20 highly similar sequences with average sequence diversity of 4 % and mostly intact reading frames. These data imply that this clade might represent the outcome of a more recent amplification event (Baucom et al. 2009a).

The Ty3-*gypsy rt* clones of quinoa also exhibited significant levels of sequence heterogeneity, but these were lower than of Ty1-*copia rt* sequences, with an average diversity of 30 %. Higher *rt* sequence similarity resulted in recovery of one large clade corresponding to *Dell/Tekay*-like lineage, subdivided into several smaller very similar sequence groups (average similarity range from 83 to 93 %). These data are consistent with a model of evolutionarily recent amplification of a relatively small subset of closely related ancestral elements. The remaining sequences form *Reina* lineage most likely represent older retrotransposition events as evidenced from disrupted reading frames and higher nucleotide diversity. Overall lower sequence divergence of Ty3-*gypsy rt* in comparison to Ty1-*copia rt* elements was also reported previously in other species groups, e.g., *Sorghum* or *Orobanche* (Muthukumar and Bennetzen 2004; Park et al. 2007).

Most of the *rt* sequences analyzed in this study were present in the quinoa genome in low copy numbers, with the exception of the clones pCquty118, pCquty119 (*Tork/Angela* lineage) and pCquty99 (*Retrofit/Ale* lineage). Similarly, in the genomes of species of the genus *Beta* closely related to *Chenopodium,* several abundant Ty1-*copia* [Cotzilla1 (*Sire/Maximus*), SALIRE1 (*Tork/TAR*), Weber et al. 2010] as well as Ty3-*gypsy* retroelements were reported [Bongo3 (*Del/Tekay*), Beetle7 (*CRM/CR*), Bingo1 (*Reina*), Weber et al. 2013; Elbe3 (*Athila*), Wollrab et al. 2012]. The copy number of individual LTR retroelement families in plant genomes can vary greatly from very few copies to thousands of copies (Du et al. 2010). It has been hypothesized that most of the LTR retrotransposon families, regardless of their age, might contain very low numbers of intact elements (repository) as shown for soybean and maize (Baucom et al. 2009b; Du et al. 2010). Conversely, only a few retroelement families that successfully amplified in the genome in the recent past have been represented in genomes in high copy number with mostly intact reading frames (e.g., only 5 % of LTR retrotransposon families identified in the maize genome were very abundant; Baucom et al. 2009b). Different evolutionary trajectories of various lineages of retroelements (cycles of amplification and deactivation/removal) influence dynamics of genome size and genome rearrangements (Vitte and Bennetzen 2006) generally biased toward genome size increase. Polyploidy, particularly allopolyploidy, might change the dynamics of retrotransposon populations of parental genomes and might promote either genome downsizing (often affecting also retrotransposable element populations) or genome size increase (Hawkins et al. 2006; Renny-Byfirld et al. 2011), and might also play a role in shaping retrotransposon population in quinoa.

Ty1-*copia* elements, and to a smaller extent also Ty3-*gypsy* elements, were preferentially localized in pericentromeric regions of *C. quinoa* chromosomes. These regions are often heterochromatin rich (also in quinoa) and might be preferentially targeted by retroelement insertions. Not only were the most abundant quinoa *copia*-*rt* clones pCquty119/pCquty118 preferentially located in pericentromeric heterochromatin, but also the overall retrotransposon density was higher in pericentric regions (as evidenced from FISH with pooled *rt* regions, excluding pCquty119/pCquty118) than in subterminal chromosomal regions. Similar pericentromeric localization was reported for two Ty3-*gypsy* retrotransposons *Beetle* (*CRM/CR* lineage) and *Bingo1* (*Reina*) in *B. vulgaris* chromosomes (Weber et al. 2013). However, other Ty3-*gypsy* elements showed different chromosomal organization in *Beta*. For example, *Bongo3* (*Del/Tekay* lineage) was clustered along all chromosomes with reduced hybridization signal intensity in some of centromeric regions (Weber et al. 2013). SALIRE elements (Ty1-*copia, Tork/TAR*) isolated from *B. vulgaris* revealed dispersed hybridization patterns with hybridization signals strongly reduced in the centromeric and pericentromeric regions (Weber et al. 2010). The pericentromeric localization of LTR retrotransposon was reported for several other species, particularly these with small genomes, e.g., *Arabidopsis thaliana* (Brandes et al. 1997), tomato (Wang et al. 2006), or soybean, where approximately 87 % of the LTR retroelements were found in the recombination-suppressed pericentromeric regions (Schmutz et al. 2010). Selective targeting within heterochromatin might benefit the mobile element by escaping negative selection arising from insertion into genes in distal regions of the chromosome (Gao et al. 2008; Neumann et al. 2011). On the other hand, certain families of retrotransposons commonly found in centromeres of various plant groups were also shown to contribute to centromere function (Neumann et al. 2011). More detailed analyses of composition of retroelement populations in quinoa using next generation sequencing (NGS) should provide more definite answers concerning the potential role of retrotransposons in centromere function.

Two highly repetitive clones bearing similarity to fragment of RNase H (of Ty3-*gypsy* retrotransposons), co-amplified from the quinoa genome, were localized in chromosomes in a pattern similar to highly amplified *rt* fragments of the *copia* type (pCquty119). These sequences might represent another family of retroelements underrepresented in *rt* amplicons, rearranged elements, or alternatively they might represent a new type of dispersed repeat that originated from retroelements. Current data do not allow inference of the genomic origin and fate of these RNase H-like sequences.

Our study provides the first insight into the composition of the dispersed repetitive DNA fraction of the polyploid quinoa genome and the results of this study contribute the preliminary information important for planning of further studies of retrotransposons in quinoa genome (e.g., isolation of full-length LTR retrotransposons). Both Ty1-*copia* and Ty3-*gypsy* retrotransposons recovered from PCR amplification were highly heterogeneous and represented most of the known evolutionary lineages (mainly *Tork/TAR* and *Retrofit/Ale* for Ty1-*copia*; *Dell/Tekay* and *Reina* of *Chromovirus* lineage for Ty3-*gypsy*). Despite obvious limitations of the method, the combination of PCR amplification, cloning, Southern blotting, and FISH analyses of *rt* fragments of retrotransposons used in this study provide the first glimpse into diversity and organization of these elements in *C. quinoa*. Recent advances in NGS have began to provide more comprehensive evidence for differential and taxon-specific dynamics of various families of repetitive DNA, including abundant *copia* and *gypsy* elements in plants (Macas et al. 2007, 2011; Hribová et al. 2010; Kelly and Leitch 2011). Comparative genomic analyses of putative parental diploids and allopolyploids indicate differential, lineage-specific expansion and removal of various families of retrotransposons during their evolution, resulting in genome size fluctuations (Hawkins et al. 2006; Renny-Byfirld et al. 2011; Parisod et al. 2012). Phylogenetic analyses of whole genus *Chenopodium* implemented with GISH analyses (B. Kolano unpubl.) strongly support allotetraploid origin of *C. quinoa*. Thus, to understand the patterns of its genome evolution, the comparison of the repetitive fraction of quinoa genome with its putative ancestral species will be necessary. Further analyses of the repetitive DNA fraction in *C. quinoa* will be built on data presented in the current study and should provide more detailed insight into organization and evolution of the tetraploid quinoa genome, especially in comparison to diploid progenitors.
